# Development and Characterization of a Reverse Genetic System for Studying Dengue Virus Serotype 3 Strain Variation and Neutralization

**DOI:** 10.1371/journal.pntd.0001486

**Published:** 2012-02-28

**Authors:** William B. Messer, Boyd Yount, Kari E. Hacker, Eric F. Donaldson, Jeremy P. Huynh, Aravinda M. de Silva, Ralph S. Baric

**Affiliations:** 1 Division of Infectious Diseases, Department of Medicine, University of North Carolina School of Medicine, Chapel Hill, North Carolina, United States of America; 2 Department of Epidemiology, Gillings School of Global Public Health, University of North Carolina, Chapel Hill, North Carolina, United States of America; 3 Department of Microbiology and Immunology, and Southeast Regional Center of Excellence for Biodefense and Emerging Infectious Diseases Research, University of North Carolina School of Medicine, Chapel Hill, North Carolina, United States of America; University of Rhode Island, United States of America

## Abstract

Dengue viruses (DENV) are enveloped single-stranded positive-sense RNA viruses transmitted by Aedes spp. mosquitoes. There are four genetically distinct serotypes designated DENV-1 through DENV-4, each further subdivided into distinct genotypes. The dengue scientific community has long contended that infection with one serotype confers lifelong protection against subsequent infection with the same serotype, irrespective of virus genotype. However this hypothesis is under increased scrutiny and the role of DENV genotypic variation in protection from repeated infection is less certain. As dengue vaccine trials move increasingly into field-testing, there is an urgent need to develop tools to better define the role of genotypic variation in DENV infection and immunity. To better understand genotypic variation in DENV-3 neutralization and protection, we designed and constructed a panel of isogenic, recombinant DENV-3 infectious clones, each expressing an envelope glycoprotein from a different DENV-3 genotype; Philippines 1982 (genotype I), Thailand 1995 (genotype II), Sri Lanka 1989 and Cuba 2002 (genotype III) and Puerto Rico 1977 (genotype IV). We used the panel to explore how natural envelope variation influences DENV-polyclonal serum interactions. When the recombinant viruses were tested in neutralization assays using immune sera from primary DENV infections, neutralization titers varied by as much as ∼19-fold, depending on the expressed envelope glycoprotein. The observed variability in neutralization titers suggests that relatively few residue changes in the E glycoprotein may have significant effects on DENV specific humoral immunity and influence antibody mediated protection or disease enhancement in the setting of both natural infection and vaccination. These genotypic differences are also likely to be important in temporal and spatial microevolution of DENV-3 in the background of heterotypic neutralization. The recombinant and synthetic tools described here are valuable for testing hypotheses on genetic determinants of DENV-3 immunopathogenesis.

## Author Summary

Infectious virus clones are valuable tools for studying how changes in viral genetic codes affect viral biology. Dengue virus is the most important mosquito-borne virus worldwide, yet dengue virus infectious clones have historically been challenging to make and manipulate, making it very difficult to study the variety of genetic changes observed in dengue viruses. Here we describe the construction of a panel of five dengue virus serotype 3 (DENV-3) clones using a novel strategy not previously employed in dengue research. This strategy uses genetic fragments and synthesized genes to introduce genetic changes while minimally affecting the virus. Each of the five recombinant clones was designed to express genetically distinct DENV-3 envelope proteins derived from strains circulating in different regions of the world. We used the recombinant viruses, coupled with DENV-3 sera from geographically defined human cases, to study the impact of E variation on neutralization outcomes. Our data demonstrate that the recombinant viruses varied significantly in their neutralization outcomes, depending on sera. While it has long been presumed that infection, and vaccination, with one serotype confers lifelong protection against all variants of that serotype, our results indicate that this assumption requires a more rigorous assessment by the DENV community.

## Introduction

Dengue virus (DENV) is an enveloped (+) RNA virus in the family Flaviviridae, genus *Flavivirus* transmitted by the bite of *Aedes* spp. mosquitoes. DENV occurs throughout the tropics and subtropics and infects approximately 50 million individuals annually. There are four distinct serotypes, DENV-1–DENV-4. While prospective studies have found that most infections are asymptomatic, a proportion of infected persons will develop symptoms that include fever, rash and myalgia [1], [2] with 2% or less developing the severe disease syndromes of dengue hemorrhagic fever/dengue shock syndrome (DHF/DSS) [2], characterized by hemorrhage, vascular leakage, hypovolemia and, if untreated, shock, end organ failure and death [3]. Approximately 15,000–30,000 persons die annually from DHF [1]. DHF/DSS has been classically associated with secondary infections that occur in the context of pre-existing heterotypic immunity - leading to hypotheses that DHF/DSS is an immune mediated phenomenon driven by cross-reactive DENV antibodies and/or or DENV specific CD8+ T-cells (for reviews see: [4], [5]. Virus genotype also clearly plays an important role in severe disease pathogenesis, as. Multiple studies of DENV molecular epidemiology have found associations between circulating virus genotype and disease severity [6]–[12]. However, the genetic basis of these virulence differences has not been deciphered.

One of the fundamental barriers to DENV vaccine development has been concern that a DENV vaccine must be broadly protective against all four serotypes or recipients will risk secondary-like infection and the severe disease associated with naturally acquired secondary infection. Most vaccine trials have assessed protection against all four serotypes using prototype or vaccine related virus isolates [13] and studies need to address the degree to which intra-serotype genotypic differences may affect antibody-mediated immunity to any of the DENV serotypes, including DENV-3. While genotype specific genetic differences are scattered across the viral genome, the envelope glycoprotein (E) is the main target of neutralizing human antibody and is one logical first choice for assessing the genetic basis of differential antibody mediated neutralization of DENV-3 infection. The E glycoprotein exists as a homo-dimer with 3 distinct domains – I, II, and III [14]–[17], that, on the mature DENV virion, are arranged in a flat herringbone pattern with icosahedral symmetry [14]. Domains I (EDI) and II (EDII) are linearly discontinuous and fold to form a central eight-stranded ß barrel (domain I) with a lateral protrusion (domain II) that contains the highly conserved fusion loop required for virion fusion with endosomes. Domain III (EDIII) is a continuous peptide that extends from domain I and forms an Ig like fold that is believed to be the ligand for an as yet unidentified cellular receptor.

A successful dengue vaccine should induce broadly protective antibodies against all geographic variants of each serotype. The dengue community has long held that primary infection with one serotype confers long-lasting immunity to that serotype, irrespective of the infecting virus genotype. This is based principally on early human challenge trials [18] and multiple observational studies that have shown that, within a particular region, re-infection with the same serotype generally does not occur. Geographic partitioning of DENV genotypes significantly limits our understanding of the role of strain variation in protective immunity, as the vast majority of DENV infected persons in endemic regions never travel to regions where other DENV genotypes are circulating. Several recent findings indicate that genotypic variation may be important in immunity. Recent studies of DENV-3 strain variants using recombinant proteins and whole virus have found that neutralization mAbs raised against one DENV-3 genotype have limited neutralization activity against heterologous genotypes [19]–[22]. After primate vaccination, studies with polyclonal immune sera have also demonstrated variable neutralization of DENV3 strains [23]. In a study of pediatric dengue cases in Thailand, investigators observed significant differences in the ability of sera to neutralize reference and clinical strains of DENV3 [24]. A recent WHO report on dengue neutralization testing highlighted the need for evaluating vaccine induced immune responses using contemporary strains representing the different serotypes and genotypes of dengue [25].

DENV-3 consists of four distinct genotypes: I, II, III and IV, each originally associated with a specific geographic region [26]. Currently genotype I and II are circulating in Asia, genotype III is circulating in the Indian subcontinent, Africa and Latin America, and genotype IV appears to have been displaced but occurred throughout the Caribbean in the 1960s and 70s [7], [26]–[31]. Here we described the construction of a four-fragment DENV-3 infectious clone platform and a panel of isogenic DENV-3 recombinant viruses that captures DENV-3 E glycoprotein genotypic heterogeneity. While our approach is novel for flaviviruses, human coronavirus (CoV) investigators have used a similar system to introduce large, synthesized genomic elements into recombinant viruses to investigate genetic variability in CoV biology and pathogenesis (see [32]–[35] for examples). The CoV systems are a powerful tool for expanding understanding of genetic differences in CoVs and the application to Flaviviruses may prove similarly powerful. We subsequently tested the isogenic recombinant viruses against a panel of immune sera from people exposed to primary or secondary DENV infections. These data demonstrate a role for natural epitope variation in virus neutralization and escape. The molecular clone should also prove to be a valuable tool for studying a variety of other aspects of DENV-3 biology, pathogenesis, immunopathogenesis, epitope mapping and evolution.

## Materials and Methods

### Ethical Statement

The Institutional Review Board of the University of North Carolina at Chapel Hill approved the protocol for recruiting and collecting blood samples from people. Written informed consent was obtained from all donors.

### Tissue Culture

Vero E6 cells (ATCC CRL-1586) were maintained in MEM supplemented with 10% FCS (Gibco), non-essential amino acids (Gibco), L-glutamine (Gibco) and Anti-Anti antibiotic mix (Gibco) at 37°C in 5% CO_2_. C6/36 cells (ATCC CRL-1660) were maintained in MEM supplemented with 5% FCS, non-essential amino acids, L-glutamine and Anti-Anti at 28°C in 5% CO_2_.

### DENV3 Molecular Clone Strategy

**Figure 1:**
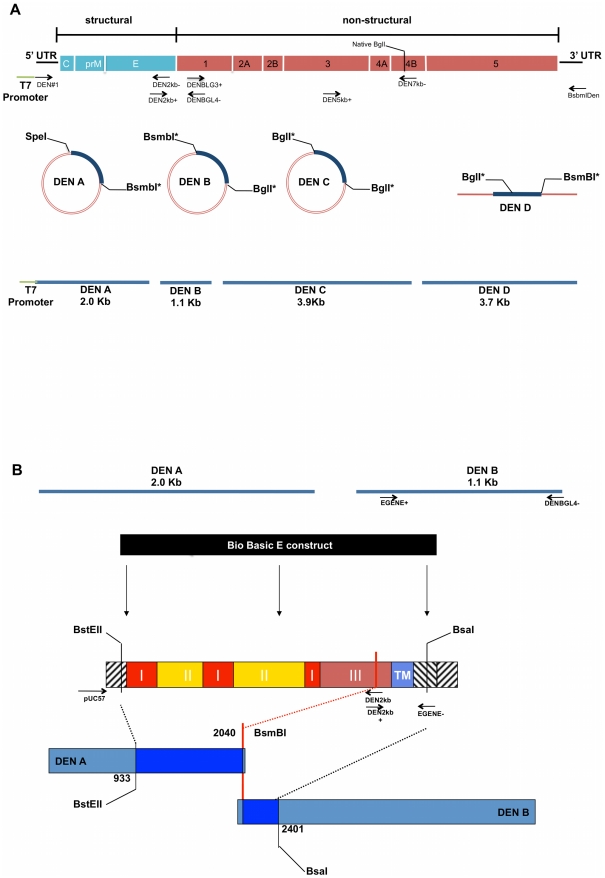
Design of DENV3 infectious clone and chimeras. Panel A provides a schematic representation of the DENV genome, divided into structural and nonstructural genes. Arrows indicate primer name and approximate primer locations and orientation on the genome. These primers were used to amplify the different cDNA fragments as well as adding appropriate, terminal restriction enzyme recognition sequences. The T7 promoter is located at the 5′end of primer DEN#1. Primer pairs that generate complete fragments are aligned opposite one another. The clone was propagated in in three circular and one linear plasmid. DENV fragments within each plasmid are highlighted in blue. The final fragments assembled to generate the clone are represented at the bottom of the figure as blue lines. Lengths and restriction site locations are approximate and supported by exact primer sequences, positions and PCR fragment sizes as noted in Text S1. Panel B illustrates the strategy used for E gene construct insertion. The top figures represent the DEN A and B fragments, which encode for the E protein. Arrows indicate approximate locations of primers EGENE+ and EGENE− used to silently introduce a BsaI recognition sequence into the 5′ end of fragment DEN B. The Bio Basic E construct in black represents the synthesized E gene, and the schematic below it illustrates approximate arrangement of E gene domains and restriction sites. Roman numerals indicate E domains; TM = transmembrane region. Arrows indicate primer names, approximate location and pair orientation used to introduce restriction enzyme recognition sequences Lengths and positions shown are approximate. The E construct is amplified for insertion into the DEN A fragment with primers pUC57 and with DEN2kb− and for the B fragment with primers DEN2kb+ and EGENE−. After sequence confirmation, constructs and DEN A and B fragments are digested with indicated enzymes, desired fragments gel purified and subjected to ligation to generate new DEN A and DEN B fragments containing E constructs. *Type IIS restriction endonuclease.

The cloning strategy for the DENV-3 clone is illustrated in Figure 1A, and based on strategies employed with CoVs to circumvent sequence instability problems in *E.coli* [36], [37]. The clone parent is a 1989 Sri Lankan DENV3 isolate (genotype III) designated UNC3001 (submitted to GenBank). To isolate the DENV-3 sub-clones, reverse transcription was performed with AMV reverse transcriptase (Roche) and oligodeoxynucleotide primers according to the manufacturer's recommendations using primer BsmbIDen. Following cDNA synthesis, the cDNA was amplified by PCR with Expand Long TAQ polymerase (Boehringer Mannheim Biochemical) with cycle settings based on the size of the amplicon. The Dengue genome was amplified from cDNA and cloned as a set of four fragments (Figure 1 and Text S1). The first fragment, A, was PCR amplified using primer set DEN#1 and DEN2kb−. These primers created a T7 RNA promoter at the 5′ end of the fragment and a BsmBI restriction site at its 3′ end, respectively. The PCR product was gel isolated (Qiagen QIAquick Gel Extraction Kit) and then cloned into the pCR-XL TOPO cloning vector (Invitrogen).

The second fragment, B, was amplified using primers DEN2kb+ and DENBGL4−. The DEN2kb+ primer introduced a BsmBI site that allowed for the directional ligation of fragments A and B (Figure 1 and Text S1). The DENBGL4− primer introduced silent changes in the Dengue genome between nucleotides (nt) 3150 and 3160 to create a unique BglI site without altering the amino acid sequence. Fragment C was amplified with primers DENBLG3+ and DEN7kb−. This primer set duplicated the BglI site at the 3′end of the B fragment and a naturally occurring BglI site at nt 7031. The PCR amplicons for both fragments B and C were gel isolated and cloned into the pCR-XL TOPO cloning vector.

Fragment D was amplified with primers DEN5kb+ and BsmBIDen. This PCR product, which went from approximately nt 5100 to the 3′ end of the Dengue genome, contained two BglI sites; one at nt 7032 and the other at nt 10186. The BglI site at nt 10186 was removed using overlapping PCR. Two amplicons – 3′ and 5′, were generated using primers Dengue15 and Den10198 and primers Den10166 and BsmBIDen, respectively. These two amplicons were joined in an over-lapping extension PCR reaction. The resulting product was digested with SapI and ligated to SapI digested DEN D fragment. This final cDNA fragment, which now had the BglI site at 10186 knocked out, was gel isolated and cloned into the Big Easy v2.0 Linear cloning vector (Lucigen).

Four to six clones of each fragment were sequence verified. The four DEN cDNAs were isolated from plasmids and directionally ligated to create a full-length cDNA of the dengue viral genome. This full-length cDNA contained only the introduced nucleotide changes, all of which were silent, and could be transcribed with T7 polymerase (Ambion). This RNA produced infectious dengue virus when electroporated into Vero E6 cells.

To construct E glycoprotein variant clones (Figure 1B), synthesized envelope genes (nucleotides 913–2416 of the Dengue genome) were delivered in puc57 plasmids (Bio Basic). The portion of these envelope genes that needed to be inserted into the A plasmid, was PCR amplified with either a puc57 forward or reverse primer and the Den2kb− primer (Text S1). These products were digested with BstEII and BsmBI and ligated into the A plasmid which had been digested with the same enzymes. Dengue B plasmids containing the envelope variants were generated by first PCR amplifying the synthetic genes with the Den 2kb+ primer and primer EGENE− (Text S1) and the parent B fragment with primer EGENE+ and DENBGL4− (Text S1). These products were then digested with BsaI and ligated together. Finally, the ligations were gel purified and cloned into the pCR-XL TOPO cloning vector.

To replace the parent clone prM/M gene with a genotype I prM/M gene, RNA from our lab stock genotype I virus UNC3043, was reverse transcribed with random hexamers and the cDNA was PCR amplified with primers Dengue01+ and Denv900. The resulting amplicon was digested with BstAPI and PflMI. This product was ligated into the DEN A plasmid corresponding to the Indonesia 1982 genotype I E gene that had been digested using the same enzymes. The resulting plasmid DEN A was sequence verified and used to construct the genotype I recombinant virus.

### Recombinant Virus Recovery

Each plasmid was transformed and propagated in *E. coli* TOP10 competent cells (Invitrogen) and grown on LB plates with selective antibiotics (A, B, and C containing plasmids selected with kanamycin, D with chloramphenicol) at 28.5°C for 24 hours. Individual colonies were picked, screened and sequenced. The plasmids were subsequently grown to high concentration in selective LB, plasmid purified (Qiagen Mini-Spin Kit) and digested as follows according to manufacturers instructions: DEN A with SpeI (NEB) followed by calf intestine phosphotase (NEB) and BsmBI (NEB) yielding a 2.0 kb fragment; DEN B with BglI (NEB) and BsmbI yielding a 1.1 kb fragment; DEN C with BglI yielding a 3.9 kb fragment; and DEN D with BglI and BsmbI yielding a 3.0 kb fragment. Fragments were gel-isolated (Qiagen Gel Extraction Kit) on 0.8% agarose gel, mixed in equivalent copy number and ligated with T4 ligase (NEB) overnight at 4°C. Full-length transcripts of DENV-3 cDNA constructs were generated in vitro as described by the manufacturer (Ambion, Austin, Tex; mMessage mMachine) with the following modifications: For 30-µl reaction mixtures supplemented with 4.5 µl of a 30 mM GTP stock, resulting in a 1∶1 ratio of GTP to cap analog and incubated at 37°C for 2 hours. Vero cells were grown to 75% confluence, trypsinized and resuspended in RNAse free PBS at 10^7^ cells/ml. RNA transcripts were mixed with 800 µl of the Vero cell suspension in an electroporation cuvette, and four electrical pulses of 450 V at 50 µF were given with a Bio-Rad Gene Pulser II electroporator. The transfected Vero cells were seeded at 5×10^6^/ml in 75-cm2 flask and incubated at 37°C for 4 days. Two to five ml of supernatant from electroporated Vero cells were passaged on day 4 to 75% confluent uninfected Vero cells in a 75 cm^2^ flask. Fresh media was added to a final volume of 15 ml. Seven day supernatants were harvested, supplemented to 30% FBS, clarified by centrifugation and frozen at −80°C or passaged serially to amplify a working virus stock.

### Envelope Gene Design

At the time this study was initiated, there were 164 unique, full-length DENV-3 envelope genes available in Genbank, and these sequences were added to 11 Sri Lankan DENV-3 sequences from our laboratory. The 175 envelope amino acid sequences were aligned using ClustalX version 1.83 [38], and one representative sequence was selected for each DENV-3 genotype. The representative sequence was chosen based on amino acid conservation within the genotype cluster, with sequences closest to consensus with no outlier amino acids selected as the representative. Representative sequences chosen were: Genotype I Indonesia 1982 (GenBank accession# DQ401690.1); Genotype II Thailand 1995 (GenBank accession# AY676376); Genotype III Cuba 2002 (GenBank accession# AY02031); and Puerto Rico (PR) 1977 (GenBank accession# AY146761). All viruses used in the subsequent experiments were passage three propagated in Vero cells. All passage three clones were sequence verified using previously described methods [39].

### Growth Curves

To assess viral replication kinetics, each of the DENV-3 clones was inoculated in triplicate onto 95% confluent monolayers of Vero or C6/36 cells in 6 well plates at a multiplicity of infection (m.o.i) of 0.01 ffu/ml. Cells were incubated at either 37°C for Vero or 27°C for C6/36 cells under maintenance media conditions for the cell line for 60 minutes, after which the innocula were removed and cells washed twice in 3 ml of PBS. Each monolayer was covered in a total volume of 5 ml media. After 60 min, 200 ul of cell supernatant, designated as the Day 0 sample, was taken in duplicate with equal volume media replaced. Samples were supplemented with 30% FCS, clarified by centrifugation and stored at −80°C. Samples were taken in the same manner every 24-hrs for 6 additional days. Virus titers were determined as described below.

### Primary and Secondary Sera

Sera were collected from adult volunteers with histories of DENV infection [40] and one anonymous donor with dengue infection confirmed by serology (sample 109). Sera were characterized by flow cytometry at UNC [41], PRNT_60_ at the NIH, Bethesda, MD, or PRNT_90_ at CDC San Juan to confirm past exposure to primary or secondary DENV infections and also to identify the serotype responsible for primary infections. We note that we cannot establish the infecting virus genotype of our experimental sera on neutralization patterns alone. However, only genotypes I, II, and III are currently circulating, and our samples almost certainly capture genotype II (Thailand) and III (Latin America) based on donor travel history.

### Virus Titration and Focus Reduction Neutralization Test (FRNT)

The FRNT procedure is based on a method previously described by Whitehead [42]. Briefly, twenty-four well plates were seeded with 5×10^4^ Vero cells in MEM supplemented with 5% fetal bovine serum (FBS) and grown for 24 hours. Growth media was removed. For virus titration, virus stocks were diluted serially ten-fold from 10^−1^ to 10^−6^ and 200 ul of each dilution added to individual wells. After 1 hr incubation on a rocker at 37°C, the wells were overlaid with 1 ml 0.8% methylcellulose in OptiMEM (Gibco) supplemented with 2% FBS (Cellgro) and antibiotic mix (Gibco Anti-Anti). Plates were incubated 5 days at 37°C, 5% CO_2_. On day 5, overlay was removed, cells washed with PBS, fixed in 80% methanol and either stored at −80°C or developed. To develop plates, fixed monolayers were blocked for 10 minutes with 5% instant milk PBS, followed by incubation with anti-flavivirus MAb 4G2 diluted 1∶1000 in blocking buffer for 1 hr at 37°C. Wells were washed with PBS and incubated with horseradish peroxidase (HRP) conjugated goat anti-mouse Ab (Sigma) diluted 1∶500 in blocking buffer for 1 hr at 37°C. Plates were washed once in PBS and foci developed by the addition of 100 ul of TrueBlue HRP substrate (KPL). Foci were counted on a light box and viral titers calculated by standard methods. For FRNT, MAbs or human sera were serially diluted five-fold from starting dilutions of 1∶5 or 1∶10. Each dilution was mixed with approximately 30 focus forming units (ffu) of virus to a final volume of 200 ul, incubated for 1 hour at 37°C, 5% CO_2_ and added in triplicate to 24 wells plates and processed as above. Mean focus diameter was calculated from ≥20 foci/clone measured at 5× magnification.

### Software and Statistics

Multiple alignments were performed using ClustalX version 1.83 [38] and phylogenetic trees of the envelope protein sequences were conducted using Mr. Bayes version 3.12 (Huelsenbeck JP, 2001). Briefly, 175 amino acid envelope sequences were imported into ClustalX and the alignment was performed using default parameters. Structural models of the informative sites were generated using MacPymol (Delano Scientific) and the crystal structure of DENV-3 envelope (PDB 1UZG) [16]. Mean focus sizes were compared by one-way analysis of variance (ANOVA) followed by Dunnett's test for multiple comparisons. Growth curve and FRNT counts were entered into Graphpad Prism (Version 5.00 for OSX, GraphPad Software, San Diego California USA, www.graphpad.com). FRNT_50_ values were calculated by sigmoid dose-response curve fitting with upper and lower limits of 100 and 0 respectively. All error bars show 95% confidence intervals unless otherwise specified. Mean FRNT_50_ values were compared by one-way ANOVA followed by Tukey HSD multiple comparison test with significance level alpha (P) set at <0.05.

## Results

### Construction of the Parent and Isogenic Envelope Glycoprotein (E) Variant Clones

**Figure 2:**
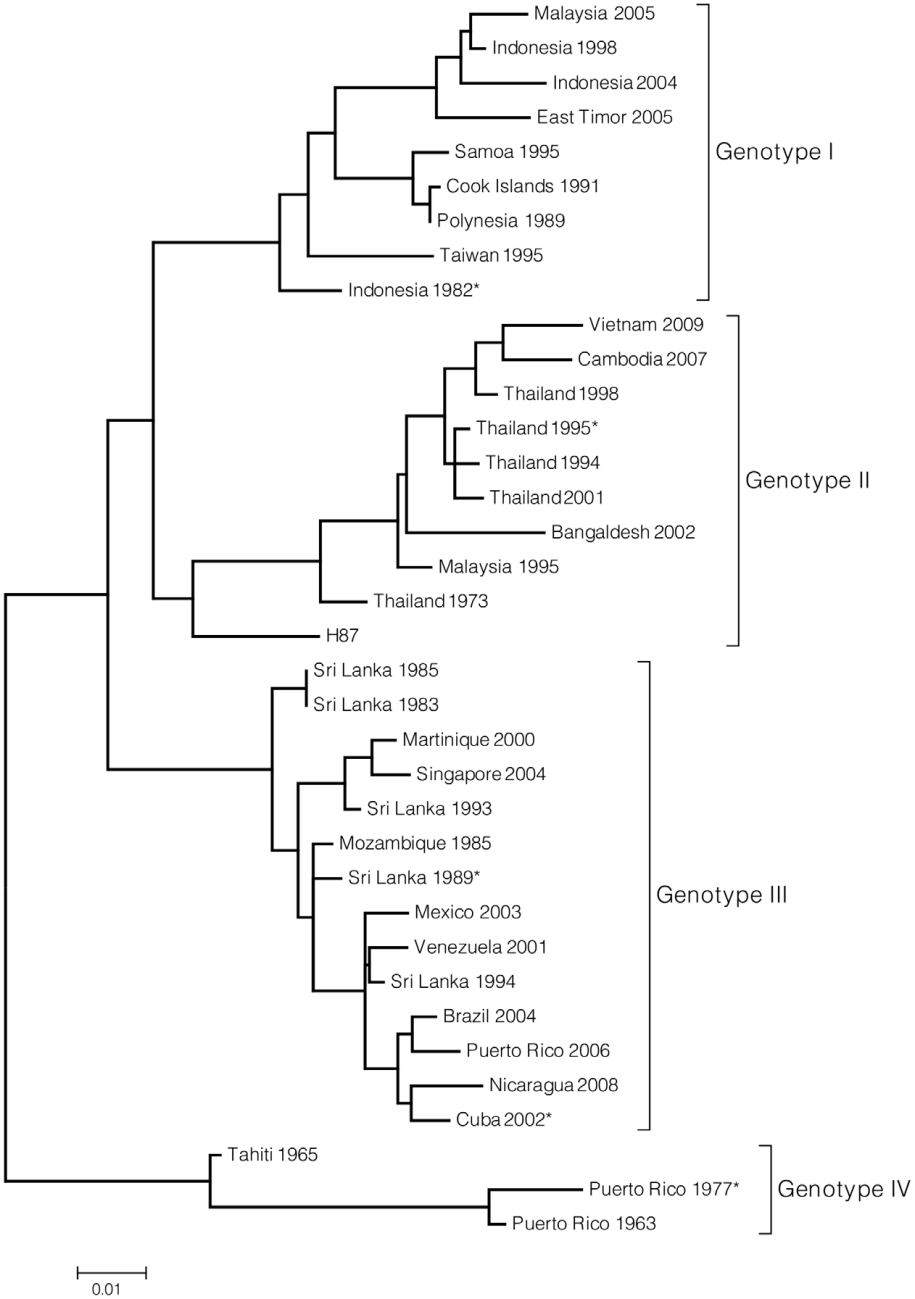
Phylogenetic relationship of DENV-3 viruses. The phylogenetic tree illustrates genetic relatedness of DENV-3 virus genotypes, including those viruses from which representative E genes were synthesized. This tree is meant to display DENV-3 diversity but does not include all 175 sequences used to evaluate the genetic variability of the DENV-3 E gene. Instead, representative sequences from each genotype were selected for inclusion in the tree. The tree was constructed using Maximum Likelihood method based on the Tamura-Nei model [66]. The tree is drawn to scale, with branch lengths measured in the number of substitutions per site. The analysis involved 37 nucleotide sequences. There were a total of 1479 positions in the final dataset. Evolutionary analyses were conducted in MEGA4 [67]. *Parent virus for E gene variants.

The parent DENV-3 clone is a genotype III variant isolated from a Sri Lankan DF patient in 1989 (Figure 2) (See materials and methods). Full-length flaviviruses genomes have been previously described as unstable and toxic in traditional *E. coli* clone systems [43]–[46]. To disrupt the putative toxic regions and facilitate creation of chimeric DENV-3 clones, the genome was cloned into segmented, sequential fragments. The fragments and junctions in the final platform were chosen to through multiple trials to maximize insert and plasmid stability in *E. coli*. Clone junctions were based on type IIS restriction enzyme sites (BsmBI and BglI) (Figure 1A) that allow directional assembly into full-length cDNAs as described in Materials and Methods. After digestion and purification of individual cDNAs, the full-length cDNA was assembled by *in-vitro* ligation, transcripts were electroporated into cells and recombinant viruses were recovered from first passage Vero cell culture supernatant. Sequence analyses verified indicator mutations within the cDNA clone fragments and no nucleotide mutations were detected in the entire genome of the recombinant virus after three passages in Vero cells (data not shown).

To evaluate the role of DENV3 E protein sequence variation on antibody interactions, representative E genes from genotype I, II, III and IV viruses (Figure 2, Text S1) were selected from 175 published DENV-3 sequences. Each E gene was selected to represent a genotype whose sequence most closely matched a consensus E sequence generated for each genotype. Genotype I is a 1982 Indonesia isolate, genotype II is a 1995 Thailand isolate, genotype III a 2002 Cuba isolate, and genotype IV a 1977 Puerto Rico isolate. A total of 32 informative sites were identified across the representative genotypes (Materials and Methods), forming nine clusters on the surface of the E glycoprotein, relatively evenly distributed through domains I, II and III (Text S1).

To generate clones that would allow testing of variable neutralization, these representative sequences were synthesized by Bio Basic and inserted into the parent clone background, replacing the parent E gene (Figure 1B). Three of the four variant clones were successfully recovered with correct replacement of the E gene alone. One variant, however, Indonesia '82 (genotype I), required the replacement of the parent SL '89 genotype III preM/M gene with a genotype I preM/M gene, supporting earlier studies that co-evolutionary changes in preM/M may be essential for efficient E gene function in select instances [47]. Full-length sequencing of all passage three recombinant virus clones used throughout these experiences found only one nucleotide mutation in one of the five clones, a silent C to T pyrimidine transition mutation at genomic position 7043 in the genotype I virus.

### Focus Formation

Because some DENV clinical isolates do not reliably form plaques on Vero cell monolayers, viral growth on Vero cell monolayers was instead characterized through focus formation (see Materials and Methods). All five clones formed foci on Vero cell monolayers. The parent clone, SL '89 (III) and Cuba '02 (III) produced moderate sized and relatively uniform foci after 5d growth on a Vero cell monolayer (Table 1). Clones with Indonesia '82 (I) E genes produced marginally smaller foci, while Thailand '95 (II) and PR '77 (IV) foci were markedly smaller than those formed by the parent clone (Table 1). The striking difference in plaque phenotype underscores the importance of structural proteins in basic viral biology, and may be due to either E gene differences in the virus envelope or prM-E mismatch in the virus clones, though identifying the particular genetic differences causing the phenotype is beyond this paper's scope.

**Table 1:** Mean focus size for parent virus and chimeric clones.

Clone	focus diameter (mm)*	95% CI
SL '89 (parent virus)	1.09	1.04–1.15
SL '89 (arent clone)	1.03	0.91–1.15
Indonesia '82 (I)	0.92	0.85–0.99
Thailand '95 (II)	0.84	0.72–0.96
Cuba '02 (III)	1.09	0.97–1.21
Puerto Rico '77 (IV)	0.73	0.66–0.80

*At least 30 foci were measured for each clone.

### Growth Kinetics

**Figure 3:**
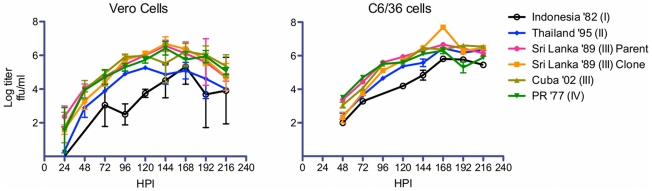
Recombinant dengue virus growth kinetics in tissue culture. Vero (figure 3A) and C6/36 (figure 3B) cells were inoculated at a multiplicity of infection (m.o.i) of 0.01 FFU. Cell culture supernatants were harvested at indicated times and the virus released from the infected cells was quantitated by immunofocus assay. Points show geometric mean titer (GMT) calculated from triplicate titrations. Error bars indicate 95% confidence intervals for each GMT. For Vero cells no focus forming units were observed at 0 hrs and for C6/36 cells no focus forming units were observed at 0 hrs and 24 hrs.

The growth kinetics of the panel of recombinant viruses were characterized in mammalian Vero cells and C6/36 mosquito cells, both of which are a commonly used for DENV propagation and quantification. Both cell lines were infected with the parent and clones at a multiplicity of infection (MOI) of 0.01 FFU/cell and grown for 216 hours. In Vero cells, the growth curves for the parent virus and the five clones were similar, with all preparations producing focus-forming virus after 24 hours and peak viral titers achieved between 120 hours and 168 hours (Figure 3A). Peak log viral titers ranged from 6.68 log FFU/ml for the parent clone to 5.10 log FFU/ml for the genotype II clone. Early growth was slower in the genotype I Indonesia recombinant virus, but ultimately reached peak titers equivalent to the other recombinants. Growth kinetics in C6/36 cells were similar to those in Vero cultures except that virus was not detected until 48 hrs post infection (Figure 3B). Peak titers were generally similar, though the parent clone had a single peak log titer of 7.70 log FFU/ml that was significantly higher than the other virus samples. The remaining peak titers ranged from 6.30 log FFU/ml to 6.66 log FFU/ml and did not differ significantly. The genotype I Indonesia clone did show slower kinetics than the other clones, particularly early in infection (Figure 3B). Overall, inter-genotypic E variability had minimal impact on the viruses' growth in tissue culture.

### Human Polyclonal Sera Neutralization

To assess the role of DENV E glycoprotein variation on viral neutralization by human polyclonal sera, the isogenic clones were tested against a panel of late convalescent (>2 years) human anti-DENV primary and secondary sera collected from individuals in North Carolina who had been infected during foreign travel [40](Table 2 and Text S1). The majority of the neutralization tests were repeated in independent experiments, with highly reproducible FRNT_50_ values (Text S1). The original infecting virus is not known for any of these sera.

**Table 2:** Summary of human primary homotypic, primary heterotypic and secondary sera used to in neutralization experiments.

Serum	Infecting Serotype	Location	Year infected	Year collected
003	DENV-3	Thailand	2001	2005
005	DENV-3	Puerto Rico	2000	2005
011	DENV-3	El Salvador	1998	2005
033	DENV-3	India	2005	2009
103	DENV-3	Nicaragua	1995	2009
105	DENV-3	Thailand	2002	2009
109	DENV-3	Sri Lanka	ND	2010
118	DENV-3	Nicaragua	2008	2010
009	Secondary	India and Sri Lanka	2000	2005
006	DENV-1	endemic areas*	1992	2005
031	DENV-2	South Pacific	1997	2005
102	DENV-4	Honduras	2007	2009

Infecting serotype was previously determined by PRNT against reference WHO strains DENV1 WestPac-74, DENV2 S-16803, DENV3 CH-53489, and DENV4 TVP-360 (Text S1). Location and year refer to where and when the traveler acquired the DENV infection. Serum is the patient identifier. ND = No data.

*Endemic areas included Latin America, South Asia and Southeast Asia.

**Figure 4:**
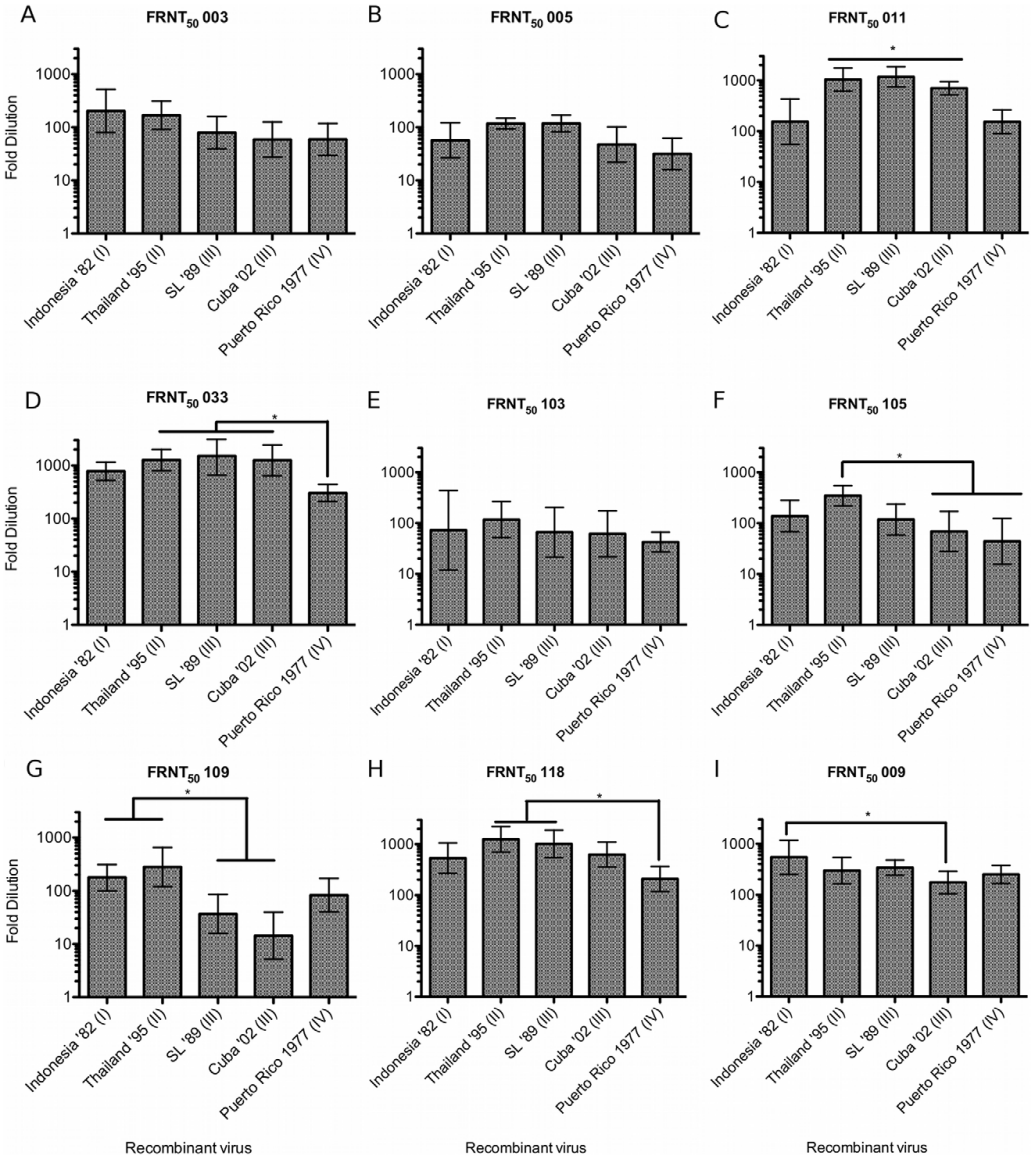
Mean FRNT_50_ values for homotypic primary and secondary sera. Homotypic primary (anti DENV-3) sera (Figures 4A–4H) or secondary serum (Figure 4I) FRNT_50_ titers against each of the E variant isogenic clones. Each serum sample is identified above the graph. Serum histories are summarized in Table 2 and Text S1. Fold-dilution of serum is on the Y-axis and each clone is identified on the X-axis. Columns show GMT FRNT_50_ values calculated from FRNT done in triplicate. Error bars indicate 95% confidence intervals. Columns connected by horizontal lines indicate groups of clones that did not have statistically significantly different FRNT_50_ values (P<0.05). Connecting bars indicate individual or groups of clones that differed statistically (P<0.05) by Tukey's HSD. Sera 003 (Figure 4A), 005 (Figure 4B) and 103 (Figure 4E) did not have significantly different titers against the recombinant viruses.

Eight primary anti-DENV-3 serum samples were tested against the parent and isogenic recombinant viruses with variable E genes from the different DENV-3 genotypes (Text S1). The clones did not show differential neutralization patterns against three of the sera; 003, 005 and 103 (Figure 4A, B, and E). Serum sample 003 was taken from a traveler who acquired a primary DENV-3 infection in Thailand. FRNT_50_ titers for 003 ranged from 1∶59 for Cuba'02 (III) to 1∶203 for Indonesia '82 (I) (Text S1). Serum sample 005 was taken from a traveler who acquired a primary DENV-3 infection in Puerto Rico. Calculated FRNT_50_ were similar to those observed for 003, with titers ranging from a low titer of 1∶31 against PR '77 (IV) to a high of 1∶118 against the Sri SL '89 (III) clone and Indonesia '82 (I) (Text S1). Serum sample 103 was from a traveler infected with DENV-3 in Nicaragua in 1995. FRNT_50_s ranged from a low 1∶42 (PR '77 (IV)) to a high of 1∶117 for Thailand '95 (II) (Text S1). While these FRNT_50_ values are consistent with most accepted cutoffs for true homotypic neutralization, they are consistently low, with six of the fifteen clone titers in this group less than 1∶60 (Text S1).

**Figure 5:**
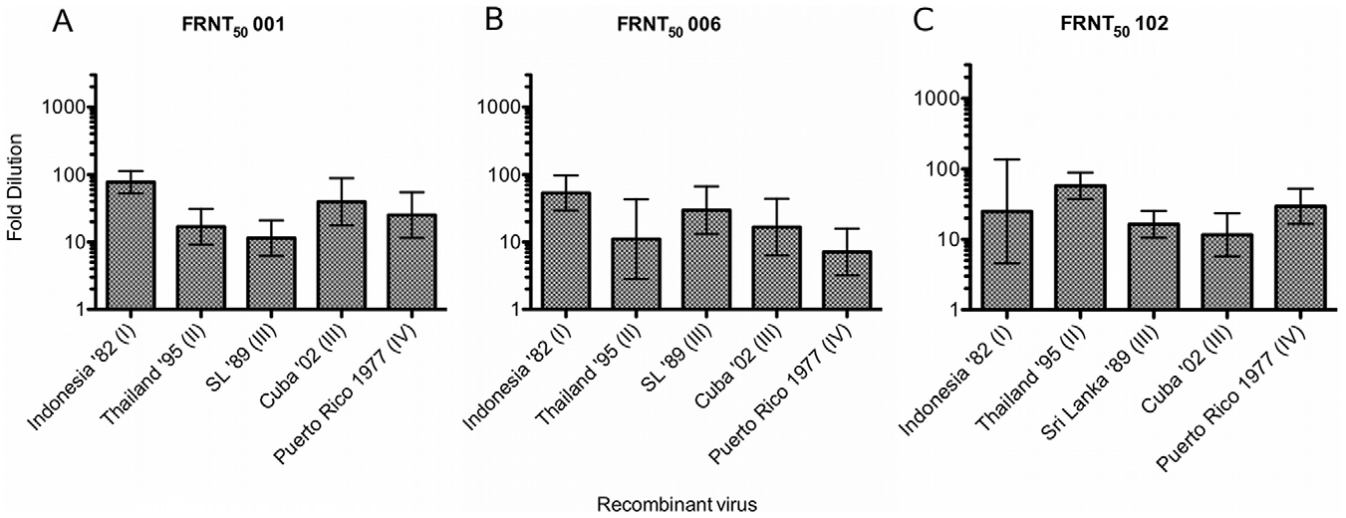
Mean FRNT_50_ values for heterotypic primary sera. Each serum sample is identified above the graph. Serum 001(Figure 5A) is from a primary DENV-2 infection in; Serum 006 (Figure 5B) is from a primary DENV-1 infection; Serum 102 (Figure 5C) is from a primary DENV-4 infection. Serum histories are summarized in Table 2 and Text S1. Fold-dilution of serum is on the Y-axis and each clone is identified on the X-axis. Columns show GMT FRNT_50_ values calculated from FRNT done in triplicate.

More importantly, we found significantly variability neutralization profiles against the five recombinant viruses neutralized with the five remaining homotypic sera tested (Figures 5C, 5D, 5F, 5G and 5H). Though serum sample 011, from an El Salvador infection, neutralized all five clones, we found a 9-fold difference (P<0.05) between the calculated lowest and highest neutralizing titers, with a low neutralizing group consisting of Indonesia '82 (I), 1∶133, and PR '77 (IV) 1∶157 and a second, high neutralizing group included the remaining clones Cuba '02 - 1∶701, Thailand '95 - 1∶1091 and SL '89 -1∶1172 (Text S1). Serum sample 033 (Figure 4D), from an infection in India, was similarly potent, with four of the five clone titers greater than 1∶780, but with the PR '77 (IV) recombinant virus again showing a significantly lower neutralization titer at 1∶304 (Text S1). Against samples 105 (Figure 4F) and 118 (Figure 4G), from infections in Thailand and Nicaragua respectively, the neutralization titers differed five (105) and six (118) –fold between recombinant viruses expressing Thailand '95 or PR '77 E glycoprotein (P<0.05)(Text S1). The most extreme neutralization differences between the clones were seen using serum 109 (Figure 4F), from a Sri Lanka donor. This serum, with titers of 1∶177 and 1∶280, efficiently neutralized the Indonesia '82 and Thailand '95 clones respectively, while the genotype III clones were neutralized at much lower dilutions of 1∶40 (Sri Lanka) and 1∶15 (Cuba) (Text S1). Thus, we observed significant variation in neutralization across DENV-3 genotypes for five of the eight primary homotypic sera tested.

Human anti DENV secondary sera are known to be broadly neutralizing across serotypes, and we would expect it to show relatively high and broad FRNT_50_ values and resist intra-genotypic variability. To test this assumption, each of the five clones were tested against 009, serum from a patient who had a secondary DENV infection, in India or Sri Lanka in 2000. All of the clones were efficiently neutralized at relatively high titers, though the highest (Indonesia '82) and lowest (Cuba '02) did differ significantly (Text S1, Figure 4H), though this difference was less than three-fold.

Heterotypic primary anti-DENV serum may have low-level serotype-cross neutralizing activity, and in one study was shown to be protective for heterotypic infection in some cases [48]. To assess the role of E glycoprotein variation in heterotypic cross-neutralization, the clone panel was tested against representative primary anti-DENV-1, -2, and -4 sera (Table 2, Text S1). Sample 001 was collected after a primary DENV-2 infection acquired in Sri Lanka in 1996. 001 had low level but detectable FRNT_50_s that ranged from 1∶11 to 1∶78 (Figure 5A, Text S1). Serum 006 FRNT_50_ titers ranged from 1∶7 to 1∶54 (Figure 5B, Text S1), and sample 102, collected after a DENV-4 infection in Honduras, had a similarly scaled FRNT_50_ range of 1∶12 to 1∶58 (Figure 5C, Text S1). However, while repeat FRNT against the clone panel with homotypic sera yielded highly reproducible neutralization titers, repeat FRNTs were not reproducible for heterotypic sera (Text S1), significantly limiting any conclusions that might be drawn from variable heterotypic neutralization.

## Discussion

CJ Lai *et al.* described the first full-length infectious DENV clone for DENV-4 isolate 814669 (isolated from a patient in the Dominican Republic in 1981 [49]) in 1991 [46]. At that time, the authors noted the full-length DENV cDNA was unstable in *E. coli.* This was overcome by using a two-fragment system that divided the toxic genomic regions. Subsequent DENV-2 New Guinea C [45] and DENV-4 West Pacific '74 clones [44] employed similar fragment based strategies to overcome genomic stability problems, though a single plasmid DENV-2 clone has also seen considerable use ([50]–[52] for examples).

Blaney *et al.*, using the DENV-3 clinical isolate Sleman '78, described the first, and, until now, only, DENV-3 infectious clone in 2004 [53]. Though based on a full-length cDNA plasmid, successful propagation of the plasmid DNA required inserting a 30 nt linker region containing termination sequences in each of the forward and reverse open reading frames near the E/NS1 junction. To date, the parent Sleman '78 clone has principally been used as a backbone for vaccine candidates [43], [54], [55].

Clearly, instability and toxicity have been the principle challenges of developing tractable DENV infectious clones. The smaller DENV cDNA sub-clone platform we employ offers several advantages. The individual fragments are highly stable in *E coli* and they can be manipulated individually without affecting distant sites on the genome and allow for fragment re-assortment between DENV strains. The type IIS restriction enzymes *Bgl*I and *Bsmb*I generates unique 5′ and 3′ overhangs and prevents spurious self-assembly of the sub-clones, a technical problem with all palindromic cutting restriction enzymes [56]. Finally, multiple mutations can be incorporated simultaneously into separate fragments, circumventing iterative mutation and sequencing of the entire molecular clone and allowing for reassortment of fragments.

With the exception of the genotype I E gene, the parent molecular clone backbone was receptive to heterotypic E sequences. We suspected that genotype specific prM/M-E interactions between the genotype III parent prM/M and genotype I E accounted for failure to recover viable genotype I E chimera. Genotype I prM/M differs from genotype II, III, and IV in 2 positions. The first is a histidine to lysine mutation at prM/M position 55. This region is predicted to form a strand between two parallel beta sheets that interacts with the E fusion loop [47] and the polymorphism at position 55 likely explains why the original genotype I clone was not viable. The second difference was a leucine to phenylalanine mutation at position 128. This polymorphism conserves the hydrophobic character of the residue, hence we think it unlikely that this mutation affected the original genotype I chimera's viability. Replacing the parent prM gene with a genotype I prM established a viable clone and argues that future constructs should include prM and E from the same genotype. However, overall, the clone platform was remarkably stable: full length sequencing of passage three of all five of the clones found only one (silent) nucleotide mutation in one - Indonesia '82 - of the five clones. The recombinant viruses grew to equivalent peak titers compared to the parent clone, though Indonesia '82 showed delayed growth kinetics in both cell lines. Different plaque phenotypes emerged with E glycoprotein changes. While chimeric construction may affect interactions between E and the non-structural proteins or directly change RNA-RNA interactions, these effects are likely subtle, given the relatively similar clone growth kinetics in tissue culture, and are unlikely to directly affect chimeric clone neutralization by polyclonal sera.

Forty years ago Halstead and others first reported variable neutralization between clinical DENV-3 isolates [57] when they observed that mouse immune sera raised against DENV-3 strain H-87 poorly neutralized low passage wild-type DENV-3 isolates from Thailand. The authors hypothesized that the observed differences in neutralization were due to within serotype antigenic differences. Shortly thereafter, Russell *et al.* reported similar findings for human immune sera [58]. They found that both human convalescent sera and mouse hyper-immune sera against Tahitian and Caribbean DENV-3 poorly neutralized H-87 and a Thailand 1965 clinical isolate with differences in 50% hemagglutination inhibition (HI) titers varying by more that 10-fold. The authors argued that the different titers were evidence of genetic subtypes within DENV-3, at the time a novel idea, although the genetic basis for this variable phenotype was unclear. The authors also argued that Caribbean strains would be poor vaccine candidates because of their antigenic properties did not elicit broadly neutralizing homotypic antibodies. Despite this early observation of variable neutralization within serotypes, the phenomenon remained largely unexplored, in part because few tools existed to isolate antigenic variation in an otherwise stable genetic background.

More recently, Zulueta *et al.* [22], found that human sera from acute genotype III DENV-3 infections were essentially non-reactive with recombinant genotype IV EDIII but appropriately reactive with genotype III EDIII. However, this study's findings were significantly limited by the use of pooled acute human sera and binding assays, rather than neutralization assays and individual human polyclonal serum samples. In a related set of experiments, Cuban researchers tested convalescent sera collected from twenty DF and DHF cases from the 2001/2002 Cuban DENV-3 epidemic against a panel of six DENV-3 isolates collected between 2000 and 2002 [19]. The sera PRNT_50_ titers against clinical isolates from before and after that epidemic differed by nearly 10-fold, with the patients' sera more effectively neutralized virus from after the epidemic than before. However, their observed differences are based on neutralization against wild type viruses representing only genotypes III and IV and only three of the seven viruses used were sequenced. Finally, Thomas *et al.* [59], using previously characterized human DENV sera, found that PRNT_50_ titers were significantly affected by both virus strain and tissue in which the virus was propagated. While these experiments strongly hint at E gene dependent differences in polyclonal antibody neutralization, they do not directly test variability in the neutralization of isogenic DENV-3 viruses encoding clearly defined E gene differences by late convalescent sera.

Our results significantly advance both the pioneering early studies of Halstead and Russell as well as the more recent work cited above, all of which collectively argue that antigenic variability in DENV-3 genotypes significantly influences intra-serotypic neutralization responses in *in vitro* assays. With our panel of sera and E variant clones, we found both dramatically large, up to 19-fold, differences in FRNT_50_ values and FRNT_50_ titers as low as 1∶15 for homotypic sera (Text S1). Our data indicate that variation in E strongly drives these phenotypes, as all other viral proteins were isogenic.

Prospective studies of DENV transmission have found that low titer pre-existing neutralizing Ab (by PRNT) in endemic areas does not uniformly protect from homotypic infection [24], and a prospective study of maternal antibody in newborns found that 50% neutralization titers of <1∶50 are often not protective against homologous virus strains, even in endemic settings [60]. Finally, a recent human challenge study in DENV-3 vaccinated subjects found that a PRNT titer 1∶57 in one vaccinated volunteer was only partially protective, and another volunteer developed both fever and viremia with a pre-existing anti-DENV-3 titer of 1∶16 [61]. Current vaccine trials define 50% or 60% neutralization titers of >1∶10 [62], [63] or 1∶20 as evidence of immunity, potentially lower than the hypothesized protective thresholds suggested by the studies cited above. Some recent vaccine studies by Durbin *et al.* Guy *et al.* have begun to test vaccinee sera against representative genotypes [64], [65]. However, Durbin *et al.* used early convalescent sera - 42 days post vaccination, which is likely to be more broadly neutralizing is too early post-vaccination to capture the durable, long-term antibody response. *Guy et al.* similarly evaluated vaccinated vaccine sera against DENV genotypic variants, but used primate rather than human sera and the authors did not specify when the samples were collected post vaccination. The magnitude of the neutralization differences we report may be enough to lead to partial protection or loss of protection in vaccines, depending on the infecting genotype. It is also possible that, in the context of live virus vaccination, broad within serotype protection is conferred even with low titer antibodies, and that genotypic differences will not matter in the context of protection. That said, Genotype IV stands out in our experiments as relatively non-reactive with homotypic human immune sera (Figure 4B, 4E, 4F) and raises the question of whether vaccination could potentially create an immunologic “niche” in human hosts that could be exploited by sylvatic or geographically and genetically distant genotypes within a serotype.

Our findings serve as a point of departure for studying the important epitopes in the human antibody response to DENV infection, most of which have not yet been defined. Clones that selectively alter the antigenic clusters distributed across E (Text S1) will facilitate initial mapping of the epitopes responsible for differential neutralization. Ideally, identifying the key neutralizing epitopes in the human polyclonal immune response will, in turn, inform rational vaccine and possibly therapeutic monoclonal antibody design - optimizing epitopes to elicit potent neutralizing antibodies. Although only speculative, the DEN3 molecular clone may also prove invaluable for identifying epitopes and antibodies responsible for enhancing dengue infection.

## Supporting Information

Text S1 **Supporting tables and figures.**
(DOCX)
