# Supplementary material for: Development and Characterization of a Reverse Genetic System for Studying Dengue Virus Serotype 3 Strain Variation and Neutralization
Source: PLoS Negl Trop Dis. 2012 Feb 28;6(2):e1486. doi: 10.1371/journal.pntd.0001486 (PMC3289595; doi:10.1371/journal.pntd.0001486)
Supplement: Text S1 — Supporting tables and figures. (DOCX) [file pntd.0001486.s001.docx]

**Supporting Information**

**Supplemental Table 1A**. Summary of primers used in clone construction (fix primer DEN7KB-).

Position is nucleotide position on UNC 3001 (Ascession # pending). Native DENV sense or anti-sense sequence shown in red except for D:10198- and D10166+ where red indicates mutated nucleotides. N indicates random nucleotide. (-) and (+) indicate positions extending beyond the 5’ (-) and 3’ (+) ends.

**Supplemental Table 1B**. Location and overhangs of ligation productions.

Junction refers to clone fragments. Overhangs are DNA sequence for the bolded fragment under Junction.

**Supplemental Table 2**. FRNT_50_ values for human sera against the all four DENV serotypes.

Infecting serotype was previously determined by neutralization assay against reference WHO strains DENV1 WestPac-74, DENV2 S-16803, DENV3 CH-53489, and DENV4 TVP-360. Location and year refer to where and when the traveler acquired the DENV infection. Infecting serotype was determined by the neutralization profile for individual sera against all four serotypes. Fifty percent neutralization was determined by FACS assay at UNC except where indicated.

*PRNT_60_ performed at NIH

**PRNT_90_ performed at CDC San Juan

**Supplemental Table 3A**. Summary of FRNT_50_ values for clones against homotypic human polyclonal sera.

FRNT_50_ vaues are reported as a mean fold-dilution.

**Supplemental table 3B**. Summary of FRNT_50_ values for clones against heterotypic polyclonal sera.

FRNT_50_ values are reported as a mean fold-dilution

**Supplemental Figure 1.**

Correlation between independent neutralization assays for homotypic sera against the clone panel. Each point plots the first experiment FRNT_50_ (X axis) vs. second experiment FRNT_50_ (Y axis) for a given serum sample against one clone. First and second experiment FRNT_50_ values were highly correlated. N=28, R^2^=0.88, P<0.0001. Dotted line shows 95% confidence interval for regression line.

**Supplemental figure 2**.

Correlation between independent neutralization assays for homotypic sera against the clone panel. Each point plots the first experiment FRNT_50_ (X axis) vs. second experiment FRNT_50_ (Y axis) for a given serum sample against one clone. There was no correlation between first and second experiment FRNT_50_. N=10, R^2^=0.012, P=0.7596. Dotted line shows 95% confidence interval for regression line.

Supplemental Figure 3.


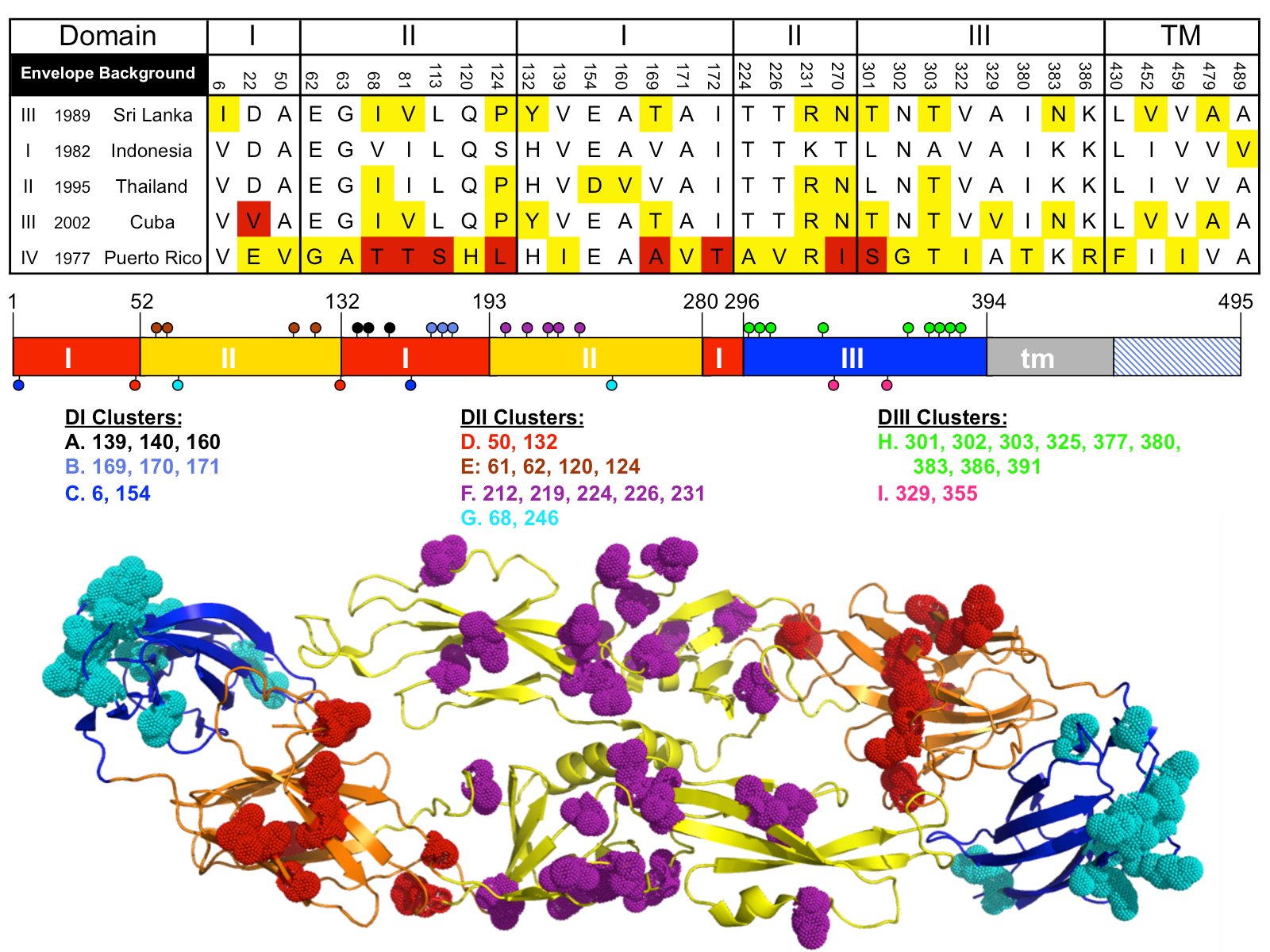


Supplemental Figure 3. Summary of DENV-3 E gene informative amino acid positions and domains. The table summarizes sites of amino acid variation are shown by domain and position within the E protein, indicated as headers across the top of the table. Colors represent differences at each position, with yellow indicating one difference and red indicating the second difference per column. Variable sites that were predicted to be exposed on the envelope protein structural surface were mapped onto a linear schematic of the E protein to demonstrate which amino acids are proximal to one another and form clusters that may represent important epitopes. Clusters are represented by color, alphabetical lettering, and amino acid positions that occur within the cluster. Each cluster color is noted on the linear E protein diagram by a similarly colored lollipop. Surface exposed amino acid variation was mapped onto the structural model of the DENV3 E protein, demonstrating that amino acids in all three domains change between different genotypes. Domain I, orange; Domain II, Yellow; Domain III, blue. Surface exposed variable residues occurring in: Domain I, red; Domain II, purple; and Domain III, cyan.
